# Comparing the Effectiveness and Safety of Giving Fentanyl or Ketamine Intranasally during Phacoemulsification Surgery

**DOI:** 10.31661/gmj.v12i.2921

**Published:** 2023-12-17

**Authors:** Hamidreza Shetabi, Alireza Peyman, Farzan Piudeh

**Affiliations:** ^1^ Department of Anesthesiology, Anesthesiology and Critical Care Research Center, Isfahan University of Medical Sciences, Isfahan, Iran; ^2^ Department of Ophthalmology, Ophthalmology Research Center, Isfahan University of Medical Sciences, Isfahan, Iran; ^3^ Student Research Committee, Medical School, Isfahan University of Medical Sciences, Isfahan, Iran

**Keywords:** Analgesia, Fentanyl, Ketamine, Intranasal, Phacoemulsification

## Abstract

Background:Phacoemulsification is the main method of cataract surgery in developed countries. Due to the importance of appropriate analgesia and the immobility of the participants throughout the procedure, the study aimed to assess the impact of intranasal ketamine vs. intranasal fentanyl on the quality of sedation and analgesia in phacoemulsification surgery. Materials and Methods: This double-blinded study was carried out on participants who underwent cataract surgery in Faiz Hospital, Isfahan, Iran. Eighty subjects were randomly assigned to two groups of 40 receiving ketamine at a dosage of 1.5 mg/kg intranasally (Intranasal Ketamine (INK) group) or fentanyl at 1.5 μg/kg nasally (Intranasal Fentanyl (INF) group). The drugs were administered through the nasal passage 15 minutes before the operation. The primary outcomes were a difference in the quality of sedation and pain relief between groups during the procedure and recovery unit. Secondary outcomes were cardiovascular parameters, side effects, the need for sedative rescues, and changes in vital signs. Results:During the study, 25 patients (62.5%) in the INK cohort and 19 patients (47.5%) in the INF cohort had no pain. In the INK group, 22 (55%) and in the INF group 20 (50.0%) patients achieved optimal sedation (Ramsay sedation score 4). There was no discernible disparity observed between the two cohorts in terms of the quality of sedation (P=0.071), receipt of rescue dosage of propofol (P=0.601), hemodynamic parameters (P0.05), and side effects during treatment Operation (P=0.542) and in recovery (P=0.104), patient (P=0.098) and surgeon (P=0.120) satisfaction, operative time (P=0.082), and duration of stay in recovery (P=0.110). Conclusion: Although INK was more effective than INF in reducing pain and achieving optimal sedation in cataract surgery, it was not significantly superior to INF.

## Introduction

Phacoemulsification is the most important method of cataract surgery worldwide [1]. Various anesthesia methods for phacoemulsification are available, including general, local, partial, or a hybrid of these modalities [2][3]. Intranasal use of medication provides effective, well-tolerated analgesia that can be administered faster compared to parenteral administration [4][5][6]. The nasal mucous membrane serves as a significant pathway for drug absorption, characterized by its extensive blood supply. This route has a direct influence on the brain through the olfactory plates, resulting in rapid systemic absorption and expedited drug action. Moreover, utilizing this route helps circumvent initial metabolism in the gastrointestinal tract and liver, leading to prolonged drug effects and potentially enhanced tolerability compared to intravenous administration [7]. The anesthetic effect of ketamine mainly works by inhibiting N-methyl-D-aspartate (NMDA) and by hyperpolarizing in cyclic nucleotide receptors [8]. The bioavailability of ketamine via the intranasal route is 45-55% with detectable blood levels within 2 minutes of administration, reaching peak concentrations within 30 minutes, and providing effective analgesia for up to 1 hour [9][10]. Intranasal ketamine provides swift and satisfactory analgesia in emergency department (ED) patients who have acute pain [11][12][13]. On the other hand, Fentanyl, with a bioavailability of about seventy-one percent, is the most common painkiller used through the nasal [14][15]. In intranasal fentanyl studies, a dosage of a dosage of 1.0 to 1.5 µg/kg of intranasal fentanyl has proven effective for analgesia in pediatric limb injuries. [16][17]. The analgesic effect of intranasally administered fentanyl and ketamine was compared in several studies [13][14][15][17][18][19].

Comparing the sedative and analgesic effects of INK vs. INF in the phacoemulsification procedure was our goal in this trial.

## Materials and Methods

**Figure-1:**
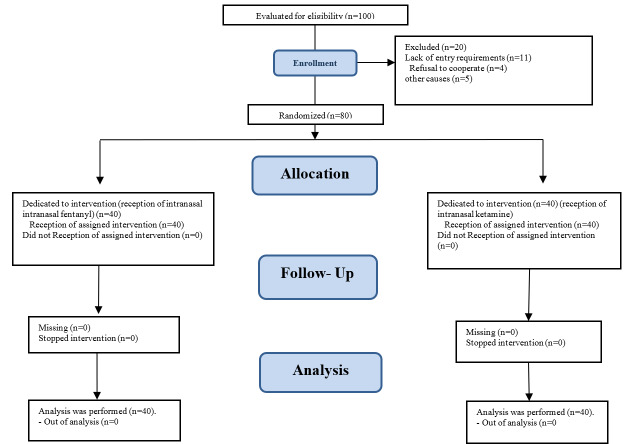
Figure 1.Consort flowchart of participants

**Table:** Table1. Population Distribution Specifications among Groups

Variables		Groups		P-value
		INK (Number=40)	INF (Number=40)	
Age in years		65.68±11.06	66.55±11.59	0.16
Weight; Kg		71.03±13.02	68.70±13.53	0.439
Sex	Female	20(50.0%)	22(55.0%)	0.823
	Male	20(50.0%)	18(45.0%)	
ASA	I	18(47.4%)	10(28.6%)	0.148
	II	20(52.6%)	25(71.4%)	

**INK:** Intranasal Ketamine;**INF:**Intranasal Fentanyl Data shown Mean±SD or n (%).

Participants

A double-blind investigation was employed at Faiz ophthalmologic Center in Isfahan on 80 subjects diagnosed with cataracts, they were randomly assigned to two groups, with 40 individuals in each. The investigation was conducted over the course of a 12-month duration, from March 2019 to March 2020 The necessary ethical approval for this study was obtained from the Isfahan Medical University ethical committee, with serial numeral IR.MUI.MED.REC.1398.129.

The research was duly registered with the Clinical Trials Centre of Iran with the ID number IRCT20170809035601N11. Prior to their participation, each patient’s informed consent was taken in accordance with the Helsinki Declaration.

Criteria for Inclusion or Exclusion to Study

The study included patients between the ages of eighteen and 75 years, who were undergoing phacoemulsification surgery and had a physical condition of I or II according to the American Society of Anesthesiologists (ASA). Excluded from the study were patients with medical impairments, pregnant and lactating mothers, individuals exhibiting a body mass index (BMI) exceeding 27 kg/m2, individuals with a history of chronic sedative or analgesic use, drug addiction, allergy to each of the study medication, participant with severe chronic obstructive pulmonary disease, and those with deviated nasal anatomy or nasal congestion.

Randomization and Blinding

A nurse employed the algorithm generated by the Random Allocation software to randomly allocate participants into two groups of 40, wherein patients would receive either ketamine or fentanyl through nasal administration. In order to ensure the blinding of surgeons, patients, and data collectors, drug syringes with identical volume and appearance were utilized.

Groups and Interventions

In the operating theater, all patients underwent standard monitoring, which included electrocardiogram (ECG), noninvasive intermittent sphygmomanometer, pulse oximetry, and capnography. through a nasal cannula, oxygen was administered with a flow rate of 3 liters per minute. All patients in both groups were given similar sedative medications ten minutes before the procedure. Propofol was administered to both groups with a bolus dose of 400 μg/kg, and a further dosage of 100 µg/kg was repeated at an intervening period of 30 seconds until reached the appropriate level of sedation (RSS score 4), The intervention pharmaceuticals utilized in the research were ready by an anesthetist who did not partake in the gathering of data. Syringe #1 was filled with ketamine at a dosage of 1.5 mg/kg (with a topmost dosage of 100 mg) for the INK group. Syringe #2 contained fentanyl at a dosage of 1.5 µg/kg (with a topmost dosage of 100 micrograms) for the INF group. The intervention drugs were administered intranasally, 15 minutes before the operation, in a volume of 2 ml, with 1 ml being delivered into each nostril. If necessary, distilled water was added to achieve the appropriate volume.

Outcomes

The primary outcomes were differences in the quality of sedation and pain relief between groups during surgery. Secondary outcomes were side effects, the need for further sedation, and changes in hemodynamic parameters. The analgesic effect of two intervention drugs was evaluated using a 10-point scale called VAS (Visual Analogue Scale). The scale ranges from Zero (signifying the absence of pain) to 10 (representing the utmost excruciating pain possible). Pain scores were categorized as follows mild pain was indicated by a score of 1-3, moderate pain by a score of 4-6, and severe pain by a score of 7-10. If the pain score exceeded 3 in either group, morphine was given at a dosage of 0.05 mg/kg. The patient’s sedation level was evaluated by the Ramsay Sedation Score (RSS), which ranged from 0 to 5 (0=anxious, 1=calm, 2=lethargic, 3=confused) but responsive to conversation, (4=unresponsive to conversation, 5=unresponsive to painful stimulation). The aim was to achieve an RSS score of 4. If the patient was still anxious, 2 mL (5 mg/mL) of propofol was available and administered to patients in both groups as a rescue sedative. In all cases, surgery was performed by the surgeon under the same operating microscope conditions. Heart rate (HR), mean arterial pressure (MAP) respiratory rate (RR), and oxygen saturation (Spo2) were meticulously monitored and documented every five minutes. Subsequently, in the recovery room, these vital signs were methodically recorded every ten minutes.

A Likert scale consisting of 5 points was employed to assess the level of satisfaction of the participant and the surgeon, spanning from "Very unhappy" to "Completely consent." When participants achieved an Aldert score of 9-10, they were ready to be discharged from recovery. Complications such as bradycardia (number of beats less than 60 per minute), hypotension (mean arterial pressure less than 60 mmHg sustained for more than 10 minutes), respiratory depression (number of effective respiratory movements less than 10 times per minute), and oxygen saturation drop to less than 92%, rapidly were treated and documented.

Statistical Analysis

Data entry was carried out using SPSS software v 24 (IBM, Armonk, NY, US). Mean with standard deviation was utilized to express variables with quantifiable characteristics, while frequency and percentages were used for variables with qualitative characteristics. To compare the qualitative variables among research groups, the chi-square test was employed for variables with quantifiable characteristics, independent samples t-test was applied. A statistical significance was designated by a P-value level further down than 0.05.

## Results

**Table:** Table2. Compare the Hemodynamic Parameters Changes among Groups

Variables		Groups		P value ^1^
		INK ((Number=40))	INF ((Number=40))	
HR; bpm	Baseline	76.6±16.2	78.6±16.1	0.125
	During surgery	76.6±12.1	77.8±13.8	0.71
	Recovery room	74.4±12.6	73.9±12.3	0.78
P value ^2^		0.27	0.11	
SBP; mmHg	Baseline	147.3±15.7	154.5±26.3	0.18
	During surgery	147.5±16.8	145.6±17.4	0.63
	Recovery room	146.9±13.9	138.7±19.7	0.68
P value ^2^		0.32	0.35	
DBP; mmHg	Baseline	89.3±9.9	88.9±10.3	0.3
	During surgery	88.8±11.5	86.9±9.7	0.45
	Recovery room	87.5±11.2	85.2±9.8	0.62
P value ^2^		0.64	0.5	
MAP; mmHg	Baseline	108.9±9.5	113.5±12.2	0.08
	During surgery	108.5±12.6	106.4±11.1	0.48
	Recovery room	107.4±11	106±12	0.91
P value ^2^		0.12	0.14	
SPO2; %	Baseline	96.5±2	97.6±1.8	0.052
	During surgery	97.8±1.2	98.3±1.8	0.18
	Recovery room	97.7±2.1	97.9±1.6	0.81
P value ^2^		0.81	0.13	

Data shown Mean ±SD 1. Statistical significance was obtained by comparing the average value of each variable among the groups at each time point. 2. The significance level is determined by comparing the average values of each variable within both groups across different time periods.

Randomization and analysis were conducted on a total of 80 participants, who were divided into two groups as depicted in Figure-1.

In the current investigation, the INK cohort comprised 20 (50%) female participants and 20 (50%) male subjects, with an average age of 65.68±11.06 years. The INF group, on the other hand, comprised 22 (55%) female subjects and 18 (45%) male participants, with an average age of 66.55±11.59 years (P-value exceeded 0.05, Table-1). There were no notable disparities observed in the average hemodynamic parameters among the two groups at each given point in time (P>0.05). Within each group, the analysis of hemodynamic parameters revealed no changes during both surgery and recovery when compared to the preoperative period. Moreover, the analysis of average alterations in hemodynamic parameters between the two groups revealed no statistically notable disparity in the observed trends (P-value exceeding 0.05, Table-2). In the postoperative care unit, it was observed that the group subjected to intranasal ketamine (INK) exhibited a less incidence of pain severity when compared to the group that received intranasal remifentanil (INF). Specifically, in the INK group, 25 patients (62.5%) reported no pain, and the remaining patients had mild pain. In contrast, in the INF group, 19 patients (47.5%) experienced no pain, and the rest reported mild pain.

In general, no significant discrepancy in pain intensity among groups was detected. (P=0.125). Specifically, the mean pain severity scores for the INK and INF groups were 0.45 and 0.14, respectively, yet this varian=0.110) the present study did not yield any statistically significant variations discernible among cohorts of INK and INF (Table-3). failed to yield statistical significance, as indicated by a P-value of 0.448.

Regarding sedation, 22 patients (55%) of the INK group and 20 participants (50.0%) of the INF group achieved optimal sedation with a Ramsay Sedation Score of 4. There were no discernible disparities observed between the two cohorts with regard to the quality of sedation (P=0.071), the need for a rescue dose of propofol (P=0.601), or the occurrence of side effects during surgery (P=0.542) and in the recovery room (P=0.104). Similarly, participants satisfaction (P=0.098), surgeon satisfaction (P=0.12), operation time (P=0.082), and recovery room time (P=0.11).

## Discussion

**Table:** Table3. Compassion Intraoperative and Postoperative Variables in the Two Groups

Variables			Groups		P value
			INK ((Number=40))	INF ((Number=40))	
Pain intensity	0		25(62.5%)	19(47.5%)	0.125
	1		7(30%)	19(47.5%)	
	2		2(5.0%)	2(5.0%)	
	3		1(2.5%)	0(0.0%)	
Mean Pain intensity			0.45 ± 0.14	0.58±0.1	0.448
Ramsay Sedation Score	2		3(7.5%)	1(2.5%)	0.071	
	3		5(12.5%)	14(35.0%)	
	4		22(55.0%)	20(50.0%)	
	5		10(25.0%)	5(12.5%)	
Rescue dosage of Propofol	0		30(75.0%)	29(72.5%)	0.601
	1		10(25.0%)	10(25.0%)	
	2		0(0%)	1(1.4%)	
Complications during surgery	Agitation		7(17.5%)	6(15.0%)	0.542
	Hypotension & bradycardia		0(0%)	1(2.5%)	
	Decreased O2sat		0(0%)	1(2.5%)	
Complications in recovery room	Dizziness Sore eyes		89.3±9.9	88.9±10.3	0.104
	Nausea		88.8±11.5	86.9±9.7	
	Itching				
	Erythema		87.5±11.2	85.2±9.8	
Patient Satisfaction			4.00±0.82	4.33±0.92	0.098
Surgeon Satisfaction			4.16±0.68	4.63±0.98	0.120
Surgery time; min			16.1±2.1	15.3±1.00	0.082
Recovery time; min			36.6±8.00	39.7±7.80	0.110

Data shown Mean±SD or n (%).

According to the findings of our study, the INK group showed lower pain frequency and intensity and better sedation achieved (RSS=4) compared to the INF group. In terms of analgesia and quality of sedation, no notable difference was observed among the two groups. Hemodynamic variables were measured during surgery and in the recovery room and there were no observable differences between the groups, and no there were no serious cases of hemodynamic abnormalities requiring medical intervention. Patient and surgeon satisfaction was slightly higher in the INF group. Our findings were aligned with previous research showing that INK and INF reduced pain scores comparably over time. For instance, Andolfoto et al. in adults with orthopedic injuries, showed that INK notably reduced clinical pain [13]. Yemen et al. conducted a similar study where INK In adults with pain, mean VAS scores in range of medium to severe decreased within 30 minutes

[14]. In a research conducted on a sample of 90 patients aged 18 years and above, it was observed that the INF cohort exhibited a significantly lower mean pain score in comparison to the IVF cohort. Conversely, the IVF group demonstrated a superior average sedation level when compared to the INF group [15]. The findings of a clinical trial conducted by Murphy et al. demonstrated that INF at a dosage of 1.5 µg/kg, is a secure and efficacious painkiller for the treatment of pain in pediatric patients within the out-of-hospital environment [16]. Nasr Isfahan et al. intranasal fentanyl 1 µg/kg, Intranasal ketamine 1 mg/kg, and intranasal normal saline were used in three groups. In the results, they found that 5 and 10 minutes after the procedure, the VAS score in the ketamine group was remarkably reduction than the INF cohort. Patient satisfaction in the ketamine group was superior to the fentanyl group [17]. Frey and colleagues conducted a study on children aged 8 to 17 years with acute and painful orthopedic injuries to the limbs, it was concluded that ketamine at a dosage of 1.5 mg/kg nasally has a suitable painkiller effect compared to fentanyl at a dosage of 2 μg/kg intranasally. Therefore, it is thought that INK can be introduced as a suitable alternative to INF in the management of pain in the context of acute organ damage [18]. In a study, the average level of satisfaction reported by patients and surgeons was higher in the IVF group compared to the INF group [19]. Yenigun et al. have reported that the effect of INK and INF in alleviating post-tonsillectomy pain among pediatric patients is similar, and they worked more effectively than paracetamol [21]. Intranasal ketamine or intranasal fentanyl is known to increase analgesia after endoscopic nasal surgery, according to a study by Hala et al. In the INF group, the occurrence of negative side effects was lower, and surgeon and patient satisfaction was higher than in the INK group [22]. In our study, the effectiveness of intranasal fentanyl and intranasal ketamine in pain relief is consistent with other studies [17][18][21][22]. In our study, side effects were slightly more in the INK in contrast to the INF during the study period. This finding is consistent with previous studies [17][18][21].

## Conclusion

Although INK was more effective than INF in reducing pain and achieving optimal sedation in cataract surgery, it was not significantly superior to INF. On the other hand, a slight increase in mild side effects was observed in INK compared to the receiving INF group.

## Acknowledgment

This article is based on the MD thesis of Farzan Piudeh. The present study’s research proposal was granted approval by the esteemed Research Council of the Faculty of Medicine, Isfahan Medical University. The authors express their gratitude to all individuals who contributed to this research.

Financial support: No financial support was provided.

## Conflict of Interest

There are no conflicts of interest.
